# Main Determinants of Supplementary Health Insurance Demand: (Case of Iran)

**DOI:** 10.5539/gjhs.v7n6p285

**Published:** 2015-04-23

**Authors:** Soraya Nouraei Motlagh, Hassan Abolghasem Gorji, Ghadir Mahdavi, Hossein Ghaderi

**Affiliations:** 1 Department of Health Economics, School of Health Management and Information Sciences, Iran University of Medical Sciences, Tehran, Iran; 2 Department of Health Services Management, School of Health Management and Information Sciences, Iran University of Medical Sciences, Tehran, Iran; 3 Health Management and Economics Research Center, Iran University of Medical Sciences, Tehran, Iran; 4 ECO College of Insurance, Allameh Tabataba’i University, Tehran, Iran

**Keywords:** bayesian logit, classification and regression trees, supplementary medical insurance

## Abstract

**Introduction:**

In the majority of developing countries, the volume of medical insurance services, provided by social insurance organizations is inadequate. Thus, supplementary medical insurance is proposed as a means to address inadequacy of medical insurance. Accordingly, in this article, we attempted to provide the context for expansion of this important branch of insurance through identification of essential factors affecting demand for supplementary medical insurance.

**Method:**

In this study, two methods were used to identify essential factors affecting choice of supplementary medical insurance including Classification and Regression Trees (CART) and Bayesian logit. To this end, Excel® software was used to refine data and R® software for estimation. The present study was conducted during 2012, covering all provinces in Iran. Sample size included 18,541 urban households, selected by Statistical Center of Iran using 3-stage cluster sampling approach. In this study, all data required were collected from the Statistical Center of Iran.

**Results:**

In 2012, an overall 8.04% of the Iranian population benefited from supplementary medical insurance. Demand for supplementary insurance is a concave function of age of the household head, and peaks in middle-age when savings and income are highest. The present study results showed greater likelihood of demand for supplementary medical insurance in households with better economic status, higher educated heads, female heads, and smaller households with greater expected medical expenses, and household income is the most important factor affecting demand for supplementary medical insurance.

**Conclusion:**

Since demand for supplementary medical insurance is hugely influenced by households’ economic status, policy-makers in the health sector should devise measures to improve households’ economic or financial access to supplementary insurance services, by identifying households in the lower economic deciles, and increasing their financial ability to pay. Moreover, insurance companies should adjust their insurance policy according to clients’ needs, household characteristics, and their incomes.

## 1. Introduction

Throughout the world, policy-makers have identified adequate access of the entire population to health services as an important issue, and the principle aim of most governments is to best organize and provide health care finances to reach universal access to essential health services (Binnendijk, Dror, Gerelle & Koren, 2013; Devlin, Sarma & Zhang, 2011).

Despite efforts made, developing societies are far from reaching universal health care cover yet (Carrin, Waelkens, & Criel, 2005; Denis Drechsler & Jütting, 2005), and the health system in these countries have failed to provide adequate access to health services and financial protection for citizens, such that many people still rely on out-of-pocket payment to provide finances for their health care needs (D. Drechsler & Jütting, 2007; Jütting, 2004; Pauly, Blavin & Meghan, 2009; Preker, Scheffler & Bassett, 2007). Therefore, sustainable tools are required to provide finances for health, in order to reduce direct payments and occurrence of catastrophic health care shocks (D. Drechsler & Jütting, 2007). Accordingly, to facilitate access to health care and protect households against poverty caused by out of pocket payments, medical insurance companies are under greater scrutiny in these countries (Carapinha, Ross-Degnan, Desta, & Wagner, 2011; Nguyen & Knowles, 2010; Spaan et al., 2012; A. Wagner & Ross-Degnan, 2009; A. K. Wagner et al., 2011). Many governments have developed policies in an attempt to cover their populations with insurance and social welfare system (Jafari et al., 2005).

However, in the majority of these countries, the volume of medical insurance services, provided by social insurance organizations is inadequate (Ferdosi & Mohammadi Zadeh, 2007). Currently, in Iran, the basic level of medical insurance commitment is inappropriate, basic medical services are not clearly defined, and even some essential services haven’t been included by universal insurance commitment in the regulations, and most medical facilities cannot be used by the insured. Thus, supplementary medical insurance is proposed as a means to improve the status of medical insurance (Dehnavieh, Rashian, & Maleki, 2011; Karami & Sadoughi, 2009).

Use of supplementary medical insurance has many advantages, so that, supporters of this insurance policy claim that this type of insurance can fill the financial gaps through keeping value for money for users, avoiding queues, avoiding poor quality of care, and problems associated with under-the-table payments, which is predominantly seen when households use compulsory social insurance schemes (Zweifel, 2005).

Undoubtedly, use of supplementary medical insurance services can satisfy a large group of the mandatory social medical welfare funds insured. On the other hand, their reduced visits to doctors and hospitals contracted by social insurance and freeing available capacities, somehow lead to improved quality of services in these sections, and greater satisfaction of the social medical insurance funds insured in the long-run (Ferdosi & Mohammadi Zadeh, 2007).

Briefly, supplementary medical insurance aims to provide the possibility to purchase expensive and unaffordable health services, and thereby create welfare for users and applicants of this insurance, possibility of use of private medical facilities for insured people, filling the gap in services and commitments of basic medical insurances, create conditions for innovation, diversity and competition in the field of medical insurance, with emphasis on people’s contributions toward providing financial resources. Thus, given the above, development of supplementary insurance is considered a necessity. However, an overview of performance of organizations providing insurance services, it seems that this part of the national health system is faced with many problems including inadequate knowledge of supplementary medical insurance and their role in national health system, lack of adequacy of services and necessary comprehensiveness, and lack of obvious boundaries between supplementary and basic medical insurance (Nyman & Barleen, 2005; Vafaee Najar, Karimi & Seydnowzadi, 2007).

Additionally, losses made in supplementary medical insurance contracts have made insurance companies hesitant in issuing such insurance contracts. For instance, in 2011 and 2012, losses made by medical insurance were 101% and 108% respectively (“Statistical report on the performance of the insurance industry, Insurance Institute (affiliated to the Central Insurance),” 2009-2012).

For this reason, this important insurance discipline, which is an industry in its infancy, has not developed much. To explore causes of low profitability of supplementary medical insurance for insurance companies and problems in supplementary medical insurance market, its supply and demand features must be identified to enable provision of solutions for improvement and enhancement, and eliminating various factors inhibiting the growth of this industry and identifying and reinforcing the effective factors paved the way for its growth. Accordingly, this article attempts to provide the context for expansion and dynamics of this important branch of insurance through identification of the most important factors affecting demand for supplementary medical insurance.

## 2. Methods

Two methods were used to classify target variable and identify essential factors affecting choice of supplementary medical insurance including Classification and Regression Trees (CART) and Bayesian logit. Study samples were based on size and method of the Statistical Center of Iran. Statistical Center of Iran runs an annual national survey with the aim to examine incomes and expenses of households of the country which is optimized annually based on recent demographic and population data. To this end, household income-expense questionnaire was designed that included information about Social characteristics of household’s members, Household’s expenditures (food & non-food), incomes and facilities and completed through direct interviews with household heads. In this survey, sampling is performed in a 3-stage cluster sampling process. In the first stage, geographical regions are selected, in the second stage, clusters, and in the last, households. In 2012, final sample size was 18541 urban households, which was used in this study. Since there are infinite factors affecting demand for medical insurance, the first step in implementation of model is to select appropriate explanatory variables for the study. According to past research and economic theories, the most important factors that affect demand for medical insurance include household relative income and socio-economic status, household health status, people’s risk-aversion intensity, and demographic factors (gender, and household size) (Kiil, 2012; King & Mossialos, 2005; Kirigia et al., 2005; H. Liu, Gao & Rizzo, 2011). To quantify household health status and assess its effect on demand for supplementary medical insurance, household medical and health expenditures are assumed to reflect their health status. In other words, the implicit assumption is lack a systematic error in predicting future health status by the household. Also, to include risk-aversion effect on demand for medical insurance, it is assumed that factors of age and education of household head contain useful information about the intensity of household risk-aversion. This issue has been indicated in other studies which are based on the theory. (Castellucci, 2008; Ioncică, Petrescu, Ioncică, & Constantinescu, 2012).

Given the above, data used in the study of variables are presented in Table 1.

**Table 1:** description of variables

Independent variables	Explanations
Employ _Num	Number of working household members
Household_ Rank	Household deciles
Household _Sex	1 for female-headed households and zero for male-headed households
Household _Education	1 for Illiterate heads, 2 for Primary, 3 for Junior high school,4 for Diploma,5 for Higher diploma,6 for Degree,7 for Masters,8 for PhD and above
Household_ size	The number of individuals in each household
Household_ income	Continuous
Household_ age	Continuous
Household_ health exp.	Continuous
Household_ accommodation ownership	2 for renting households and 1 for homeowners
Life insurance	Paid household life insurance premium

**Depended variable**	**Explanations**

Household_ Supplementary insurance	1 for households with supplementary insurance and zero for others

### 2.1 CART and Bayesian Logit Methods

One of the most well-known data mining tools which have been used extensively in various research fields is Classification and Regression Trees model. This model essentially has no pre-defined basic relation between independent and dependent variables. As a result, it is turned into a strong method for classification and prediction problems, especially, when exists a great volume of data and many explanatory variables.

The principle technique in CART in creating a decision tree is that all of data are initially centralized atop of the tree in the root node. Afterwards, this node is split into two child nodes, according to one of the explanatory variables which make the most homogeneity. Indeed, data in each child node are more homogeneous than the parent node above. This process continues for every child node till existing data within a node have the most homogeneity. Such node is referred to as the terminal node, and does not produce any further branches.

One of the most common indexes used to split classification tree is GINI Index, illustrated as follow:


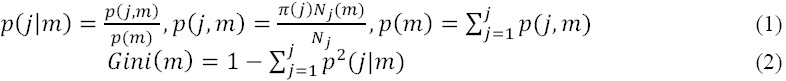


Where, J is the groups’ number, or target variables, and *π(j)* prior probability of class J, *p(j|m)* is probability of observation of class J in node m, and, *Gini(m)* represents GINI Index that shows impurity in node m.

The GINI index is calculated at each node for all variables, and a variable is selected as the separator variable, to find the minimum value of GINI(Kashani, Shariat-Mohaymany, & Ranjbari, 2012). In modeling, one of the most important stages is to identify variables with more essential role in predicting target variable. One of the classification tree outputs is variable importance index. In the classification tree with M nodes; if *S(X_j_,K)* is splitting in the K_th_ internal node base on variable *X_j_*, then, importance of this variable is the mean weight reduction in GINI impurity index. Hence, relative importance of variable is found according to the following equation:


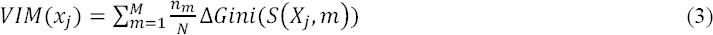


In which, ∆Gini(S(x_j_, m)) is reduction in GINI index at node m according to variable x_j_, n_m_⁄N is ratio of observations at node m, M is the number of nodes, and N is the number of observations (Pande & Abdel-Aty, 2006).

As well as classification and regression trees method, Bayesian logit was also used as a discrete choice model to determine main variables and factors affecting choice of supplementary medical insurance.

Bayesian logit model is used in cases where dependent variable is binary. Dependent variable in this model is supplementary medical insurance demand. Value of the variable for choosing and using supplementary medical insurance is 1, and for not using zero.

Unlike the classic method, where a unique value is found for each parameter of study sample, in Bayesian estimation of a regression model, posterior probability distribution of parameter under study is extracted. To extract this distribution, first, introduction of a pre-probability distribution for each parameter is required. In this method, using sample data and the introduced prior distribution, various estimates of studied parameter are created in the sample space, and using these estimates, posteriori distribution of parameters is obtained (Greenberg, 2008).

Using this model, probability of choice and demand for supplementary medical insurance is first found. Then, the marginal effect of posteriori distribution of each explanatory variable is found. The marginal effect is the change in probability of the event of dependent variable for a unit increase in explanatory variables.

To analyze data, first the required statistics were collected and entered in Excel® software for refinement, and finally, econometrics techniques were applied for calculations using R software. In Bayesian logit model, goodness of fit was assessed using Hosmer-Lemeshow test, and heterogeneity was assessed using Davidson-MacKinnon test.

## 3. Results

According to the results, about 8.04% of Iran’s population applied for supplementary medical insurance in 2012. The highest demands for this kind of insurance policy were in Qazvin, Gillan, Mazandaran, and Fars provinces respectively, and the lowest demands were in Ilam, Bushahr, and Khozestan (all among under-developed provinces). To investigate statistical relationship between household income and demand for supplementary medical insurance, percentages of households using this kind of insurance in each income stratum are presented in Table 2. Income strata from 1 to 10 show the lowest and the highest incomes, respectively. The results revealed a positive relationship between demand for supplementary medical insurance and household income.

**Table 2:** Correlation between household income and demand for supplementary medical insurance

Household expenses deciles	1	2	3	4	5	6	7	8	9	10
Percentage of households with supplementary cover	2.13	1.12	2.57	2.44	3.56	4.49	7.34	11.46	12.46	18.44
Pearson chi2 (9) = 930.1066 Pr = 0.000										

Correlation between household head education level and demand for supplementary medical insurance is shown in Table 3. As for income, household head education level was divided into 8 categories, and percentage of household demand for supplementary medical insurance was considered in each category. The results indicated a positive correlation between household head education level and demand for supplementary medical insurance, such that demand for this kind of insurance reached maximum in the highest education category. Furthermore, mean household head education level was 3.07 among households covered by supplementary insurance, and 1.75 in households not covered. In other words, higher education level led to greater demand for supplementary medical insurance.

**Table 3:** Correlation between household head education level and demand for supplementary medical insurance

Education groups	Illiterate	Primary Edu.	Junior high school	Diploma	Higher diploma	Degree	Masters	PhD and above
Percentage of households with supplementary cover	2.92	4.85	5.6	10.55	20.75	26.74	25.81	27.78
Pearson chi2 (7) = 1.3e+03 Pr = 0.000								

There is a ∩ pattern in the correlation between percentage of household’s purchasing this insurance and the age of the household head, so that ratio of households with this cover reaches maximum in 40- to 50-year-old age group. Such a pattern suggests a non-linear correlation between household head age and demand for supplementary medical insurance. This can be explained as follows: first, if insurance is considered an asset, its demand peaks in middle age, when savings and income are highest. Second, opportunity cost of wage is higher in middle age period than other periods in life, and consequently, incentive to buy insurance is greater in this period.

**Table 4:** Correlation between household head age and demand for supplementary medical insurance

Age group	<30	30-39	40-49	50-59	>60
Percentage of households with supplementary cover	1.53	6.67	9.86	9.81	8.9
Pearson chi2 (5) = 199.3622, Pr = 0.000					

**Figure 1:**
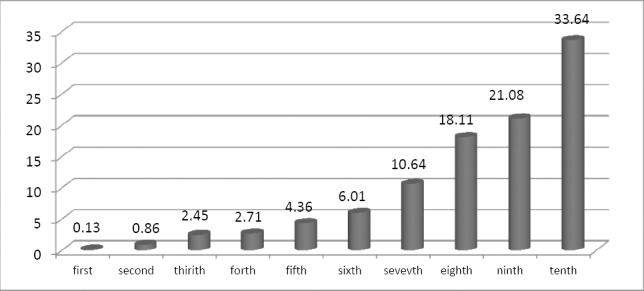
Distribution of households with supplementary medical insurance demand in different expenditure percentiles in 2012

Next, distribution of supplementary medical insurance purchasing households was examined in different income levels (expenditure deciles). It is clear from figure 1 that the highest frequency of households demanding supplementary medical insurance is observed in the highest expenditure deciles (richest groups).

Calculations of model coefficients estimates and marginal effects in Bayesian logit model are presented in Table 5. According to results in this table, demand for supplementary medical insurance increases with improved household income and socio-economic status. Household head education level showed a positive correlation with demand for supplementary medical insurance. Increased household head age, higher expected household medical expenses, and increased paid life insurance as one of the filters considered in household wealth, lead to increased demand for supplementary medical insurance. Households with female heads had greater demands for supplementary medical insurance. Increased household size and living in rental accommodation had a reducing effect on demand for this insurance policy.

**Table 5:** Mean standard deviation, and marginal effect for marginal posterior distributions

Coefficients	Mean	SD	Marginal effect
(Intercept)	-7.68	0.416	-
household_size	-0.141	0.0244	-0.0091
household_sex	0.188	0.105	0.0121
household_age	0.0846	0.0142	0.00548
Age_sqr	-.000487	0.000131	-0.000032
Household_ accommodation ownership	-0. 279	0.0887	-0.018
household_rank	0.232	0.0157	0.015
household_education	0.619	0.0662	0.0401
educatesqrt	-0.0282	0.0106	-0.00182
life_insurance	9.121e-06	1.286e-06	5.902851e-07
household_employ_num	0.00155	0.0421	0.0001

In addition to Bayesian model estimate, importance of explanatory variables, using CART method is shown in Table 6. As can be seen, the most important factors affecting demand for supplementary medical insurance include: household income, paid life insurance, and expected household medical expenses. Next to economic status of households, risk-aversion variables (age and education level) had the most importance in the demand for supplementary insurance. Meanwhile, variables of household head’s gender, house ownership, and place of residence had the lowest importance in the demand for supplementary medical insurance.

**Table 6:** Ranking of explanatory variables

explanatory variables	Relative importance
household_expenditure	100
life_insurance	75
household_healthexpenditure	66
household_age	60
household_size	25
household_education	32.7
household_employ_num	15
Household_ accommodation ownership	6.1
household_sex	5.4

## 4. Discussion and Conclusion

According to the present study results, middle-age was found the maximizing age in demand for supplementary medical insurance. Generally, the impact of age on probability of demand for medical insurance is either positive (Costa-Font & García-Villar, 2009; Taylor & Ward, 2006), or positive up to a certain age, and becoming negative or insignificant afterwards (Costa-Font & Jofre-Bonet, 2006; Doiron, Jones & Savage, 2008; Grignon & Kambia-Chopin, 2009; Rodriguez & Stoyanova, 2008). This can be explained by the high lost wage opportunity cost in middle age period, which is an extra incentive for faster recovery. Moreover, increased likelihood of sickness with aging is another incentive that decreases demand for medical insurance in younger ages. Study results indicate that demand for medical insurance maximizes with increasing education level. In addition to the direct correlation between education level and higher intensity of risk-aversion, this result can be explained by the positive correlation between education level and income, and also, correlation between education and increased awareness of benefits of low-payment regular insurance in avoiding catastrophic health expenditures (T.-C. Liu & Chen, 2002).

However, a noteworthy point is the concave relationship between demand for supplementary medical insurance and education level, which implies diminishing positive effect of education on demand. This suggests a dramatic increase in risk-aversion for every higher unit of education in low literacy levels; while this effect is less intense in higher education levels.

Study results suggest that the decision to purchase supplementary medical insurance strongly depends on household income and economic status. This variable had the greatest importance in increasing demand for supplementary medical insurance, which meant that low-income households were less likely to purchase this type of insurance policy. Yet, the principle aim of insurance schemes is to protect households against catastrophic health expenditures by offering appropriate and affordable insurance premiums. Therefore, it is necessary to conduct interventions such as payment of premium subsidies from public funds, to ensure coverage of the poor and their membership in the plan. Payment of insurance subsidies should be appropriate to income distribution in the society and local conditions. In line with the present study, several studies conducted on the demand for medical insurance indicate greater inclination for buying supplementary and private insurance among the more educated and well-off households (Bolin, Hedblom, Lindgren & Lindgren, 2010; Costa & Garcia, 2003; Grignon & Kambia-Chopin, 2009; Herring, 2005; H. Liu, et al., 2011; T.-C. Liu & Chen, 2002; Nelson, Bolen, Wells, Smith & Bland, 2004; Nguyen & Knowles, 2010; Paccagnella, Rebba & Weber, 2013; Saliba & Ventelou, 2007). One of the factors that lead to increased demand for supplementary medical insurance is number of working household members, and because of their income generation, greater numbers of these members, leads to increased available resources and household payment capabilities.

The present study results show the negative effect of household size on the demand for supplementary medical insurance However, this factor doe not significantly impact the demand for this insurance policy. The negative effect of household size has been demonstrated in a study conducted in South Africa (Kirigia et al., 2005). With increasing household size, and household income remaining constant, household per capita income is reduced. Thus, increased household size is regarded as a life standard-reducing factor.

Demand for supplementary medical insurance is formed according to expected medical expenditures. Next to household income and paid life insurance premium, this variable has the greatest importance in the demand for supplementary medical insurance, which can contain an important message for insurance providers, in relation to vulnerability of supplementary medical insurance schemes to the important phenomenon of adverse selection. To deal with this problem, supplementary insurance schemes should use such methods as incrementing risk pool, segmenting consumers basing risk categories, and insuring the whole household, instead of individual members. Cline, Mott study of demand for supplementary drug plans in elderly Medicare members showed that increased elderly drug expenditures over the previous 30 months and increased drug used by them in this period increased their likelihood of membership in supplementary drug plans (Cline & Mott, 2003). The positive effect of medical expenses on increasing demand for medical insurance is in line with previous studies (Alegria et al., 2006; Schokkaert, Van Ourti, De Graeve, Lecluyse & Van de Voorde, 2010; Yellaiah & Ramakrishna, 2012).

Although owning a house variable was of little importance in the present study, household in rental accommodations benefited less from supplementary medical insurance. This can be justified by the strong correlation between household economic status and house ownership.

In the present study, another result was confirmation of the effect of household head gender, with least importance in likelihood of demand for supplementary medical insurance. Even though, in some studies, such as those conducted in England, likelihood of demand for medical insurance was greater in male heads, due to greater affordability and inclination in men, in order to have faster access to services and avoid waiting time (King & Mossialos, 2005; Taylor & Ward, 2006). Most studies conducted on the demand for medical insurance have shown that female household heads have had a positive effect on the possibility of using medical insurance, which is in line with the present study results (Herring, 2005; Lim et al., 2007; T.-C. Liu & Chen, 2002; Nelson, et al., 2004). This result can be explained by results of experimental studies, showing women are more risk-averse than men (Eckel & Grossman, 2008). Moreover, women usually show their demand for medical facilities more than men.

Finally, it can be asserted that a better understanding of factors that make the difference in supplementary medical insurance policy among households, not only is useful in development of necessary policies to decrease differences in insurance coverage, but also effective in reducing differences in access to and use of medical care, which is strongly dependent on level of coverage.

Since households’ economic status is highly effective in the demand for supplementary medical insurance, policy-makers in health sector should take measures to improve financial or economic access of households to supplementary insurance services, including identifying households in the lower expenditure deciles, and to consider necessary measures to increase financial access and affordability of these households.

Meanwhile, it seems, insurance companies should adjust their covers with requirements of clients, household characteristics and incomes, and create incentives in employers for membership in these insurance schemes. Considering the significant effect of education level on the demand for supplementary medical insurance, providing appropriate context to increase public awareness by the government will significantly increase demand for this insurance branch.
